# Supplementary material for: Association between industry payments and prescriptions of long-acting insulin: An observational study with propensity score matching
Source: PLoS Med. 2021 Jun 1;18(6):e1003645. doi: 10.1371/journal.pmed.1003645 (PMC8205129; doi:10.1371/journal.pmed.1003645)
Supplement: S1 Text — (DOCX) [file pmed.1003645.s002.docx]

**S1 Text.**

**Data analysis plan for:**

Association Between Industry Payments and Prescriptions of Long-acting Insulin: An Observational Study with Propensity-Score Matching

# Background and Objectives

The rising cost of insulin has become one of the most important public health issues in the U.S., causing the limited affordability of this life-saving drug for patients with diabetes. Since physicians are the conduit between insulin suppliers and patients with diabetes, it is critically important to identify the upstream determinants of physicians’ prescription of long-acting insulin. Prior work has shown that payments from the pharmaceutical industry to physicians have the potential to influence clinical decision-making, but empirical evidence linking industry payments and physicians’ prescriptions focusing on long-acting insulin (the most commonly used and most costly type of insulin) has not been established.

*Aim 1*. We will examine the association between physicians’ receipt of industry payments and their subsequent prescription of long-acting insulin.

*Aim 2*. We will examine the dose-response relationship between the number of meals sponsored by the pharmaceutical industry and prescriptions of long-acting insulin.

# Data sources and Study population

We will use data from i) the CMS Open Payments data for 2016 [1], ii) the CMS National Plan & Provider Enumeration System (NPPES) database [2], iii) the CMS Physician Compare database [3], and iv) the CMS Medicare Provider Utilization and Payment database for 2017 [4]. To address temporality, we will use industry payments data in 2016 and prescription data in 2017. To ensure that physicians included in this study have the potential to prescribe long-acting insulin, we will restrict physicians to those who prescribed at least one antihyperglycemic therapy in both 2016 and 2017.

By matching physicians’ full name and the zip code, we will link the Open Payments database with the NPPES database as previous studies did [5,6]. Then, using physicians’ NPI, the merged database of the Open Payments and NPPES databases will be linked to the Physician Compare database and the Medicare Provider Utilization and Payment database.

# Variable definitions

## Outcomes

- Total claims of long-acting insulin in 2017 (30-day standardized, including refills)
- Total costs paid for claims of long-acting insulin in 2017
- Costs per claim of long-acting insulin in 2017, calculated by dividing total costs by the number of claims.

## Exposure

All non-research payments for long-acting insulin will be extracted from the Open Payments database in 2016. For a payment with multiple drugs (up to five), we will divide the amount and number of the payment equally by the number of reported drugs. The categories of payments include food and beverage, travel and lodging, speaker compensation or honoraria, and others.

## Covariates

Physicians’ characteristics including physicians’ sex, years in practice (estimated from years since graduation of medical school), and medical school graduated from will be extracted from the Physician Compare database. Medical schools will be categorized into 3 groups (ranked 1-20, 21-50, and others including all unranked and foreign medical schools) according to the research ranking of U.S. medical schools in 2017 [7]. Physicians’ specialty will be classified based on taxonomy codes in the NPPES databases.

# Statistical analyses

We will apply nearest-neighbor propensity score matching without replacement to 1:1 match physicians who received industry payments for long-acting insulin with those who did not receive these payments. The propensity scores for the receipt of payments in 2016 will be estimated by logistic regression models adjusting for physician characteristics. We will use a caliper of 0.20 SD of the propensity score and the balance between the two comparison groups will be assessed based on the standardized mean difference across covariates: successful balance, <10% [8].

Using the propensity-score matched samples, we will perform:

- Paired t-test to compare matched pairs of physicians for the total claims, the total costs, and the costs per claim for long-acting insulin in 2017.
- Paired t-test to compare matched pairs of physicians for changes in the above-mentioned outcomes.
- Sensitivity analyses: i) ordinary least squares (OLS) regression models with Huber-White robust standard errors and ii) negative binomial regression models, adjusting for the covariates used to estimate the propensity scores (to minimize residual confounding after propensity score matching).

Using the entire study cohort (before propensity score matching), we will also perform:

- Negative binomial regression models (to account for the right-skewed distribution of outcomes) adjusting for the same covariates used in the propensity score matching to examine the dose-response association between the number of meals (0, 1, 2-5, 6-10, 11-15, and 16+) received in 2016 and each outcome.
- Additional analyses: i) additionally adjusting for the total number of claims of antihyperglycemic therapies in 2016 and ii) restricting physicians to those who did not prescribe long-acting insulin in 2016.

Statistical analyses will be conducted using STATA version 15.

# References

1. Centers for Medicare & Medicaid Services. Open Payments. [cited 17 Dec 2019]. Available: https://www.cms.gov/OpenPayments

2. Centers for Medicare & Medicaid Services. NPPES NPI Registry. [cited 17 Dec 2019]. Available: https://npiregistry.cms.hhs.gov/

3. Centers for Medicare & Medicaid Services. Physician Compare datasets. In: Data.Medicare.Gov [Internet]. [cited 17 Dec 2019]. Available: https://data.medicare.gov/data/physician-compare

4. Centers for Medicare & Medicaid Services. Medicare Provider Utilization and Payment Data: Part D Prescriber Data. [cited 17 Dec 2019]. Available: https://www.cms.gov/Research-Statistics-Data-and-Systems/Statistics-Trends-and-Reports/Medicare-Provider-Charge-Data/Part-D-Prescriber

5. Tringale KR, Marshall D, Mackey TK, Connor M, Murphy JD, Hattangadi-Gluth JA. Types and Distribution of Payments From Industry to Physicians in 2015. JAMA. 2017;317: 1774–1784. doi:10.1001/jama.2017.3091

6. Inoue K, Blumenthal DM, Elashoff D, Tsugawa Y. Association between physician characteristics and payments from industry in 2015–2017: observational study. BMJ Open. 2019;9: e031010. doi:10.1136/bmjopen-2019-031010

7. Tsugawa Y, Blumenthal DM, Jha AK, Orav EJ, Jena AB. Association between physician US News & World Report medical school ranking and patient outcomes and costs of care: observational study. BMJ. 2018;362: k3640. doi:10.1136/bmj.k3640

8. Austin PC. Balance diagnostics for comparing the distribution of baseline covariates between treatment groups in propensity-score matched samples. Stat Med. 2009;28: 3083–3107. doi:10.1002/sim.3697
