# Supplementary material for: Association between industry payments and prescriptions of long-acting insulin: An observational study with propensity score matching
Source: PLoS Med. 2021 Jun 1;18(6):e1003645. doi: 10.1371/journal.pmed.1003645 (PMC8205129; doi:10.1371/journal.pmed.1003645)
Supplement: S1 Table — (DOCX) [file pmed.1003645.s006.docx]

**S1 Table.** Number (%) of physicians who prescribed long-acting insulin in 2016 and in 2017

|  | | **Number of physicians who prescribed long-acting insulin in 2016** | | |
| --- | --- | --- | --- | --- |
|  |  | **Yes** | **No** | **Total** |
| **Number of physicians who prescribed long-acting insulin in 2017** | **Yes** | 93,573 (64.3) | 9,387 (6.4) | 102,960 (70.7) |
|  | **No** | 9,925 (6.8) | 32,702 (22.5) | 42,627 (29.3) |
|  | **Total** | 103,498 (71.1) | 42,089 (28.9) | 145,587 (100.0) |
