# Supplementary material for: Association between industry payments and prescriptions of long-acting insulin: An observational study with propensity score matching
Source: PLoS Med. 2021 Jun 1;18(6):e1003645. doi: 10.1371/journal.pmed.1003645 (PMC8205129; doi:10.1371/journal.pmed.1003645)
Supplement: S2 Table — (DOCX) [file pmed.1003645.s007.docx]

**S2 Table.** Association between the receipt of industry payments for long-acting insulin in 2016 and claims of long-acting insulin in 2017 using ordinary least squares regression model adjusting for physician characteristics.^a^

|  | **Physicians who received industry payments for long-acting insulin in 2016** | **Physicians who did not receive industry payments for long-acting insulin in 2016** | **P-value** |
| --- | --- | --- | --- |
| **Claims of long-acting insulin in 2017** | | |  |
| Mean (95% CI) | 134.6 (133.2 to 136.0) | 76.7 (75.7 to 77.7) | <0.001 |
| Difference (95% CI) | 57.9 (56.1 to 59.6) | |  |
| **Costs paid for all claims of long-acting insulin in 2017** | | |  |
| Mean (95% CI) | $49,420 (48,944 to 49,896) | $26,069 (25,691 to 26,447) | <0.001 |
| Difference (95% CI) | $22,152 (21,503 to 22,803) | |  |
| **Costs per claim of long-acting insulin in 2017***^b^* | | |  |
| Mean (95% CI) | $300.5 (299.1 to 301.8) | $229.3 (227.7 to 230.8) | <0.001 |
| Difference (95% CI) | $71.2 (69.1 to 73.2) | |  |

CI, confidence interval.

^a^Adjusted for physicians’ sex, years in practice, specialty, and medical school attended.

*^b^* Estimated by [costs paid for all claims of long-acting insulin]/[number of all claims of long-acting insulin]. No claims were replaced as zero.
