# Supplementary material for: Association between industry payments and prescriptions of long-acting insulin: An observational study with propensity score matching
Source: PLoS Med. 2021 Jun 1;18(6):e1003645. doi: 10.1371/journal.pmed.1003645 (PMC8205129; doi:10.1371/journal.pmed.1003645)
Supplement: S3 Table — (DOCX) [file pmed.1003645.s008.docx]

**S3 Table.** Association between the receipt of industry payments for long-acting insulin in 2016 and claims of long-acting insulin in 2017 using negative binomial regression model adjusting for physician characteristics.^a^

|  | **Physicians who received industry payments for long-acting insulin in 2016** | **Physicians who did not receive industry payments for long-acting insulin in 2016** | **P-value** |
| --- | --- | --- | --- |
| **Claims of long-acting insulin in 2017** | | |  |
| Mean (95% CI) | 136.7 (135.4 to 138.0) | 75.8 (75.1 to 76.5) | <0.001 |
| Relative ratio (95% CI) | 1.80 (1.78 to 1.83) | |  |
| **Costs paid for all claims of long-acting insulin in 2017** | | |  |
| Mean (95% CI) | $49,063 (48,582 to 49,544) | $25,686 (25,438 to 25,935) | <0.001 |
| Relative ratio (95% CI) | 1.91 (1.89 to 1.93) | |  |
| **Costs per claim of long-acting insulin in 2017***^b^* | | |  |
| Mean (95% CI) | $301.9 (299.3 to 304.6) | $228.2 (226.2 to 230.2) | <0.001 |
| Relative ratio (95% CI) | 1.32 (1.31 to 1.34) | |  |

CI, confidence interval.

^a^Adjusted for physicians’ sex, years in practice, specialty, and medical school attended.

*^b^* Estimated by [costs paid for all claims of long-acting insulin]/[number of all claims of long-acting insulin]. No claims were replaced as zero.
