# Supplementary material for: Association between industry payments and prescriptions of long-acting insulin: An observational study with propensity score matching
Source: PLoS Med. 2021 Jun 1;18(6):e1003645. doi: 10.1371/journal.pmed.1003645 (PMC8205129; doi:10.1371/journal.pmed.1003645)
Supplement: S4 Table — (DOCX) [file pmed.1003645.s009.docx]

**S4 Table.** Association between the receipt of industry payments for long-acting insulin in 2016 and claims of long-acting insulin in 2017 using ordinary least squares regression model additionally adjusting for total claims of antihyperglycemic therapy in 2016.^a^

|  | **Physicians who received industry payments for long-acting insulin in 2016** | **Physicians who did not receive industry payments for long-acting insulin in 2016** | **P-value** |
| --- | --- | --- | --- |
| **Claims of long-acting insulin in 2017** | | |  |
| Mean (95% CI) | 93.4 (92.6 to 94.2) | 89.5 (88.8 to 90.1) | <0.001 |
| Difference (95% CI) | 3.9 (2.7 to 5.1) | |  |
| **Costs paid for all claims of long-acting insulin in 2017** | | |  |
| Mean (95% CI) | $33,597 (33,284 to 33,911) | $30,777 (30,509 to 31,044) | <0.001 |
| Difference (95% CI) | $2,820 (2,348 to 3,294) | |  |
| **Costs per claim of long-acting insulin in 2017***^b^* | | |  |
| Mean (95% CI) | $277.0 (275.5 to 278.5) | $224.7 (223.5 to 225.8) | <0.001 |
| Difference (95% CI) | $52.3 (50.4 to 54.3) | |  |

CI, confidence interval.

^a^Adjusted for physicians’ sex, years in practice, specialty, medical school attended, and total claims of antihyperglycemic therapies in 2016.

*^b^* Estimated by [costs paid for all claims of long-acting insulin]/[number of all claims of long-acting insulin]. No claims were replaced as zero.
