# Supplementary material for: Association between industry payments and prescriptions of long-acting insulin: An observational study with propensity score matching
Source: PLoS Med. 2021 Jun 1;18(6):e1003645. doi: 10.1371/journal.pmed.1003645 (PMC8205129; doi:10.1371/journal.pmed.1003645)
Supplement: S5 Table — OLS, ordinary least squares. (DOCX) [file pmed.1003645.s010.docx]

**S5 Table.** Association between the receipt of industry payments for long-acting insulin in 2016 and claims of long-acting insulin in 2017 using ordinary least squares regression model adjusting for physician characteristics, restricting physicians who did not prescribe long-acting insulin in 2016.^a^

|  | **Physicians who received industry payments for long-acting insulin in 2016** | **Physicians who did not receive industry payments for long-acting insulin in 2016** | **P-value** |
| --- | --- | --- | --- |
| **Claims of long-acting insulin in 2017** | | |  |
| Mean (95% CI) | 10.3 (9.8 to 10.8) | 6.7 (6.5 to 6.9) | <0.001 |
| Difference (95% CI) | 3.6 (3.1 to 4.2) | |  |
| **Costs paid for all claims of long-acting insulin in 2017** | | |  |
| Mean (95% CI) | $3,516 (3,331 to 3,701) | $2,169 (2,101 to 2,238) | <0.001 |
| Difference (95% CI) | $1,347 (1,147 to 1,546) | |  |
| **Costs per claim of long-acting insulin in 2017***^b^* | | |  |
| Mean (95% CI) | $100.8 (96.9 to 104.8) | $67.8 (66.2 to 69.3) | <0.001 |
| Difference (95% CI) | $33.1 (28.8 to 37.4) | |  |

CI, confidence interval.

^a^Adjusted for physicians’ sex, years in practice, specialty, and medical school attended.

*^b^* Estimated by [costs paid for all claims of long-acting insulin]/[number of all claims of long-acting insulin]. No claims were replaced as zero.
