# Supplementary material for: Association between industry payments and prescriptions of long-acting insulin: An observational study with propensity score matching
Source: PLoS Med. 2021 Jun 1;18(6):e1003645. doi: 10.1371/journal.pmed.1003645 (PMC8205129; doi:10.1371/journal.pmed.1003645)
Supplement: Attachment — Submitted filename: Response letter PLOSMED final.docx [file pmed.1003645.s011.docx]

We appreciate the thoughtful comments from the manuscript reviewers. Our responses are provided in **bold** font, along with comments from reviewers in *italics* font.

*Reviewer #1:*

*I didn't feel as though there was enough background given to clarify the ramifications of a switch to long-term insulin for both patients and health systems. My understanding (as a non-expert who has worked with similar data) is that long-term insulin could in fact translate to better outcomes for patients (and also potentially worse outcomes). Is a switch to long-term insulin always inappropriate with current guidelines? Is it possible that although costs are higher, this translates into appropriate expenditure for the improvement in patient outcomes?*

**Thank you for your careful review. We agree with the reviewer that a more detailed background would be helpful for the readers to understand the importance of this study. The current clinical guidelines indicate that long-acting insulin should be considered for patients with type 2 diabetes who do not achieve target glycemic control levels with oral agents and glucagon-like peptide 1 receptor agonists (GLP-1RA) in addition to patients with type 1 diabetes** (American Diabetes Association 2019)**. Therefore, in general, a switch to long-acting insulin is appropriate when patients with type 2 diabetes need greater glucose-lowering than can be obtained with oral agents and GLP-1RA, and inappropriate when they can achieve their target glycemic control levels without insulin.**

**Given that long-acting insulin analogs are more expensive than oral agents and human insulin—valid alternative options for some patients** (Cefalu et al. 2018; Lipska et al. 2017)**, and the pharmaceutical industry makes more profits through increased sales of long-acting insulins, there have been concerns about potential unnecessary prescriptions of long-acting insulins that are not supported by guidelines. Focusing on long-acting insulin and improve awareness on this topic is unique and important because the rising cost of insulin is a major public health issue in the US and long-acting insulin accounts for the largest share of both total Part D spending and industry marketing for diabetes care** (Inoue et al. 2020; Sumarsono et al. 2020)**. We have clarified this point as follows:**

**“Improving awareness about such associations for long-acting insulin analogs is needed because they are more expensive than other oral antihyperglycemic therapies and human insulin which could be alternative options for some patients with type 2 diabetes [9,10] and unnecessary prescriptions of long-acting insulins that are not supported by clinical guidelines [11] should be avoided to reduce the risk of hypoglycemia and save costs.” (Page 5, Paragraph 1)**

**“A study specific to insulin is important because Medicare Part D spending on insulin increased 840% from $1.4 billion in 2007 to $13.3 billion in 2017 [12,13] mainly due to the rapidly increasing trajectory of insulin prices and the number of Medicare beneficiaries using insulin in the United States [13–16]…… Specifically,** **it is critically important to investigate whether the financial relationship between physicians and pharmaceutical companies is one of the upstream determinants of physicians’ prescription of long-acting insulin—****the most commonly marketed, commonly used, and most costly type of insulin [9,19,20].” (Page 5, Paragraph 2)**

*My other main query was the interpretation of the results - in the main results the difference in claims (134 vs 77) and costs ($48k vs $26k) between the two groups is quite stark. When adjusted for total claims of antihyperglycemic medicines the difference diminishes (93.4 vs 89.5; and $33k vs $31k). This needs further discussion - this would seem to imply that the main mechanism for the increases in claims and costs comes from the industry funding influencing overall activity around antihyperglycemic therapy which doesn't seem to make sense on face value. Could patient load (i.e. number of patients with diabetes per physician) be on the causal pathway here and not adjusted for during the matching process and leading to the discrepancy between the main results and this particular sensitivity analysis?*

**Thank you for giving us an opportunity to clarify. The reviewer is correct that the observed differences diminished when we additionally adjusted for a total number of claims for antihyperglycemic therapies. We observed this phenomenon (at least partially) because the total number of claims for antihyperglycemic therapies are highly correlated with our primary outcome of interest (i.e., total claims of long-acting insulin), and including such variables in the model removed the real association between industry payments and prescriptions of long-acting insulin and biased our estimates toward the null.**

**However, we also acknowledge that the industry might have targeted physicians with a large number of claims of antihyperglycemic therapies, and therefore, we conducted analyses additionally adjusting for total claims of antihyperglycemic therapies (S4 Table). Our findings in this additional analysis, along with analysis for change in outcomes from 2016 to 2017 (Table 4) and another additional analysis restricting physicians to those who did not prescribe long-acting insulin in 2016 (S5 Table), indicate that the association was still found, although the actual magnitude could be weaker than our main findings. Moreover, the difference in cost per claim for long-acting insulin was $52.3 even after adjusting for total claims of antihyperglycemic therapies, which is substantial. We have added a paragraph highlighting this discrepancy between models with and without total claims of antihyperglycemic therapies in the revised manuscript.**

**“It is noteworthy that we found the significant association between industry payments and utilization of long-acting insulin even when additionally adjusting for total claims of antihyperglycemic therapies, or restricting the physician sample to those who did not prescribe long-acting insulin in 2016. Although the magnitude of the association was smaller than our main findings, these results, along with the results from the analyses for change in outcomes from 2016 to 2017, support the presence of direct impact of industry payments on prescriptions and costs of long-acting insulin.” (Page 16, Paragraph 2)**

**As you pointed out, the number of patients with diabetes per physician could also be a confounder between the receipt of industry payments and prescriptions of long-acting insulin (in addition to the covariates we adjusted for in our model), but such patients information was not available in the CMS data. We also have clarified this point in the limitation section.**

**“In addition, as the databases did not contain patient-level information, it was possible that patients’ characteristics such as severity of diabetes and complications might be different between groups of physicians” (Page 17, Paragraph 1)**

*P6. Paragraph 2. It wasn't clear that direct matching (i.e. exact matching of the names and ZIP) was used even after checking the two references - could you just add whether it was exact matching or a details of statistical linkage key if the info was used this way? 
Does the MPUP database record medication dispensings or prescriptions?*

**We apologize if this was not clear enough in our original submission. We conducted an exact matching of physicians’ full name and the zip code to link the Open Payments database with the NPPES database. The CMS Medicare Provider Utilization and Payment database include total claims, including refills. We have clarified these points in the Methods section.**

**“using exact matching of** **physicians’ full name and the zip code, we linked the Open Payments database that contains physician-level data on industry payments they received with the NPPES database that contains physicians’ National Provider Identifier” (Page 7, Paragraph 2)**

**“****the Medicare Provider Utilization and Payment database that contains physician-level data on drug utilization (e.g., the number of claims including refills, the aggregate cost paid for all claims, etc).” (Page 7, Paragraph 2)**

*P7, Paragraph 1. What proportion of the payments were single-drug, and what proportion associated with a multi-drug record? Were payments for more than five drugs excluded at this step or was 5 the limit that payments were adjusted for?*

**The proportion of payments for single drug and multiple drugs in our final data were 67% and 33%, respectively. In the Open Payments database, each payment lists up to five products if the payment was made in relation to any such products. In our data, only 0.1% of payments listed five products, and thus it is unlikely to induce severe misclassification bias due to payments related to more than five products, including long-acting insulin. We have added this information in the footnote in Table 2 and the revised limitation section.**

**“The proportion of payments for single drug and multiple drugs were 67% and 33%, respectively. Only 0.1% of payments were related to five products.” (Table 2, footnote)**

**“In the Open Payments database, each payment listed up to five products related to the payment, and thus we had a possibility of misclassification due to payments related to more than five products including long-acting insulin. However, such misclassification, if any, would not change our findings because 99.9% of payments listed less than five products in our final analytical sample.” (Page 17, Paragraph 1)**

*P8. Did you use the 0.2 SD of the logit of the PS, or PS on the original scale? Where is the balance information? Did you include interactions between these variables?*

**We used the 0.2 SD of the logit of the PS. A balance between the two comparison groups is described in Table 1 using the standardized mean difference across covariates. We did not include interaction terms because we already had a successful balance of covariates between the two treatment groups of physicians after the matching. We have revised the manuscript as follows:**

**“We used a caliper of 0.20 SD of the logit of the propensity score and evaluated the balance between the two comparison groups using the standardized mean difference across covariates” (Page 9, Paragraph 2)**

*What was the process of considering which physician characteristics were considered to be important confounders of the association between industry payments and long-term insulin prescribing?*

**We have included physicians’ sex, years in practice, specialty, and medical school attended based on prior literature showing the association of these physician characteristics with the receipt of industry payments and prescriptions. Although other variables such as patient characteristics (e.g., severity and complications of diabetes) could be potential confounders between industry payments and prescription of long-acting insulin, the databases did not include these variables, and therefore, we unfortunately could not control for them. We have clarified this point in the revised manuscript as follows:**

**“We used a logistic regression model to derive the propensity scores for the receipt of payments in 2016, that included physician characteristics (sex, years in practice, medical school attended, specialty) which were considered to be associated with the receipt of industry payments and prescriptions” (Page 9, Paragraph 2)**

**“Given the observational study design, it is possible that our models had residual confounding even though we adjusted for several important physician-level variables. In addition, as the databases did not contain patient-level information, it was possible that patients’ characteristics such as severity of diabetes and complications might be different between groups of physicians.” (Page 17, Paragraph 1)**

*Was there any missing data in the variables used to create the propensity scores? If so, how was this dealt with?*

**Among 146,147 physicians in the merged database of Open Payments, NPPES, Physician Compare, and Medicare Provider Utilization and Payments, we excluded physicians with missing data on years in practice (N=560). We did not apply a missing data approach such as multiple imputations because the number was small (<0.01% of the total study sample), and the missing might not have been at random given the measured covariates. We have added this number in the revised flow diagram (please see our response to your last comment). Having said that, if editors prefer, we are happy to conduct an additional sensitivity analysis accounting for the missingness of the data.**

*What was the criteria used for the categories in the trend test? Why was this used over the original value of the meals received variable?*

**In our original submission, we categorized participants into 6 groups according to the number of meals (0, 1, 2-5, 6-10, 11-15, and 16+) received in 2016 to investigate the potential non-linear relationship between the number of meals and total claims or costs of long-acting insulin. However, we also understand the reviewer’s concern, and to address it, we reanalyzed the data using the number of meals received in 2016 as a continuous variable (per five meals to calculate interpretable aRR). We found the association between the number of meals and utilization of long-acting insulin** **(claims, aRR [95% CI] = 1.241 [1.235 to 1.246]; costs, aRR [95% CI] = 1.268 [1.262 to 1.273]; aRR [95% CI] = 1.094 [1.090 to 1.099]). We have added these results in the revised manuscript.**

**“Finally, because meals are the most common and inexpensive type of industry payments,** **we examined the association between the number of meals received in 2016 (as continuous; per five meals to calculate interpretable estimates) and the total claims, the total costs, and the costs per claim for long-acting insulin in 2017, using the entire cohort prior to propensity score matching****.” (Page 9, Paragraph 3)**

**“****We found the association between the number of industry-sponsored meals for long-acting insulin received in 2016 (per five meals) and the total claims (adjusted relative ratio [95% CI] = 1.241 [1.235 to 1.246]), the total costs (adjusted relative ratio [95% CI] = 1.268 [1.262 to 1.273]), and the costs per claim (adjusted relative ratio [95% CI] = 1.094 [1.090 to 1.099]) for long-acting insulin in 2017, after adjusting for physician characteristics.” (Page 12, Paragraph 3)**

*P9. Sensitivity analyses. I assume that the negative binomial sensitivity analysis was used as cost data often has an inconvenient distribution (and this should be clarified in the methods), was the glm with sandwich errors (H-W) a way to estimate a model with a normal distribution while accounting for the matching?*

**We apologize if this was not clear enough in our original submission. As a sensitivity analysis, we fitted OLS with Huber-White heteroscedasticity-robust SE on the PS-matched sample in order to adjust for residual confounding that might not be accounted for by using the PS match. In addition, to account for the potential right-skewed distribution of residuals, we conducted an additional sensitivity analysis using a negative binomial regression (GLM) instead of OLS regression. We found that our findings were qualitatively unaffected by using these two approaches. We revised the Methods section (sensitivity analysis section) of the manuscript to clarify how we actually analyzed the data, and also showed the results of these analyses in S2 and S3 Tables in the Appendix.**

**“****Given the possible bias due to residual confounding even after propensity score matching,** **we re-analyzed the propensity score-matched samples using** **ordinary least squares (OLS) regression models with** **Huber-White robust standard errors adjusting for the covariates used to estimate the propensity scores. In addition, to address the possible model misspecification (i.e., to account for the potential right-skewed distributions of residuals), we also conducted the analysis using negative binomial regression models adjusting for the same variables.” (Page 10, Paragraph 2)**

*P10, Methods. Was psmatch2 or teffects used for the matching?
Was common support assessed? A figure of PS distribution by exposure group would be a helpful addition to the appendix.*

**We have used psmatch2 and assessed common support in our analysis. We have added a figure of the distribution of propensity scores by the two treatment groups (common support) in the appendix as suggested.**

**“The distribution of the propensity scores across the two treatment groups is shown in S2 Fig.” (Page 11, Paragraph 3)**

**S2 Fig.** The distribution of the propensity scores across the two treatment groups (common support)

*What method was used to get the estimates of claims, costs etc. per exposure group? Margins in Stata? Was this set at particular levels of the covariates, or at the average for the entire cohort?*

**We calculated the predicted value of each outcome based on the regression model using *margins command* in Stata** (Williams 2012)**. We used the actual observed values of covariates (default in *margins command*) rather than fix them at their means. We have clarified this point in the revised manuscript.**

**“After fitting regression models, we calculated the predictive value of each outcome** **under hypothetical exposure levels with the observed distribution of covariates using *margins command* in Stata” (Page 11, Paragraph 2)**

*P10, Results, Paragraph 1. Is it worth adding that the standardised differences after matching were below the target (and in fact are all below 1% different?)*

**We have added the following sentence as suggested.**

**“The two treatment groups of physicians were well balanced on all baseline covariates after propensity score matching (i.e., standardized differences were <1% for all covariates).” (Page 11, Paragraph 3)**

*P11/P12. Is it possible (given space limitations) to report the key effect estimates from the sensitivity analyses in the results? There is still a difference between the two exposures groups when there is adjustment for total claims of antihyperglycemic therapies but the magnitude of the difference is greatly reduced.*

**Thank you for your helpful comments. We have added key effect estimates from our additional analyses in the revised Results section. Please see our response to your second comment for the interpretation of the difference in the magnitude of the association between main analyses and additional analyses.**

**“The association was also observed when we additionally adjusted for the total claims of antihyperglycemic therapies (adjusted difference in claims, 3.9; 95%CI, 2.7 to 5.1; adjusted difference in costs, +$2,820; 95%CI, +$2,348 to +$3,294; adjusted difference in costs per claim, +$52.3; 95%CI, +$50.4 to +$54.3; S4 Table), and restricted the analysis to physicians who did not prescribe long-acting insulin in 2016 (adjusted difference in claims, 3.6; 95%CI, 3.1 to 4.2; adjusted difference in costs, +$1,347; 95%CI, +$1,147 to +$1,546; adjusted difference in costs per claim, +$33.1; 95%CI, +$28.8 to +$37.4; S5 Table).” (Page 13, Paragraph 3)**

*S1. Figure 1. This is an important diagram understanding how the data for the study was created. To clarify, there were ~5,000 physicians in the open payments database who weren't able to be matched to the NPPES data? Similarly, ~43,000 providers who had prescribed at least one antihyperglycaemic in 2016/2017 weren't matched to the physician Compare database? Also, there were ~60228 physicians who had received payments but then only 51,851 that had been classed as having done this in the final database - were these physicians that couldn't be linked with the NPI? I appreciate that this is already  a detailed diagram but if there is a way to include information on the numbers excluded at each step this would be very helpful, with linked administrative data the quality of the linkage process is very important for study validity and this would help me assess this aspect of the study.*

**We have revised the S1 Fig as follows, including further detailed information to clarify the flow of selecting our study sample. The matching rate of Open Payments and NPPES was close to prior literature** (Tringale et al. 2017)**. Additional 8,377 physicians (14%) were excluded from the merged data of Open Payments and NPPES when linking to Medicare data (i.e. physicians not treating Medicare beneficiaries). A total of 42,487 providers was excluded from Medicare data when linking to NPPES mainly because they were not Allopathic or Osteopathic physicians. Please let us know if you think more information is needed in this revised Figure.**

**S1 Fig.** Flow Diagram Showing Derivation of the Study Population

*-----------------------------------------------------------*

*Reviewer #2 (Joel Lexchin):*

*1.      Besides the increase in the price of insulin Medicare spending could also have increased because of more patients using insulin.*

**Thank you for raising this important point. We have revised the sentence accordingly.**

**“A study specific to insulin is important because Medicare Part D spending on insulin increased 840% from $1.4 billion in 2007 to $13.3 billion in 2017 [12, 13] mainly due to the rapidly increasing trajectory of insulin prices and the number of Medicare beneficiaries using insulin in the United States [13-16].” (Page 5, Paragraph 2)**

*2.      Page 4, 2nd paragraph, 6th line: The correct word should be "companies" not "industries".*

**We have corrected the word throughout the manuscript as suggested.**

*3.      Rather than using references 13-15 for the influence of promotion on physicians' prescribing behaviour, the authors should cite the systematic review by Spurling et al in PLoS Medicine that was published in 2010.*

**We have replaced the original citation with the following two systematic review papers.**

**1.	Spurling GK, Mansfield PR, Montgomery BD, Lexchin J, Doust J, Othman N, et al. Information from Pharmaceutical Companies and the Quality, Quantity, and Cost of Physicians’ Prescribing: A Systematic Review. PLOS Medicine. 2010;7: e1000352. doi:10.1371/journal.pmed.1000352**

**2.	Mitchell AP, Trivedi NU, Gennarelli RL, Chimonas S, Tabatabai SM, Goldberg J, et al. Are Financial Payments From the Pharmaceutical Industry Associated With Physician Prescribing? Ann Intern Med. 2020 [cited 14 Dec 2020]. doi:10.7326/M20-5665**

*4.      Besides academic detailing what other types of policy interventions are the authors proposing to regulate financial interactions between physicians and the pharmaceutical industry?*

**Given that academic detailing directly supported by payors may also suffer from a conflict of interest, one that favors the underuse of expensive but effective therapies, educational programs by unbiased third-party interests would be another option to improve physicians’ knowledge about updated information on drugs without bias. In addition, as some physicians believe that receiving industry payments does not affect their clinical practice** (Korenstein et al. 2010; Sah and Fugh-Berman 2013)**, further research on this topic to improve awareness of the physician-industry financial relationship (including studies using the Open Payments database) should be called for. We have added these points in the revised manuscript.**

**“****Although the Open Payments program under the Sunshine Act has improved transparency and raised awareness of the possible influence of physician-industry financial relationship on clinical practice for specific drugs such as opioids and cardiovascular drugs, more investigations are warranted for long-acting insulins to avoid unnecessary prescriptions of this costly and common drugs that affect millions of Americans with diabetes.” (Page 15, Paragraph 1)**

**“****future studies are needed to understand whether programs without conflicts of interest such as academic detailing [33]** **or other educational programs focusing on the follow-on insulin are effective to reduce the overall insulin costs by providing unbiased third-party information on long-acting insulins to physicians.” (Page 16, Paragraph 1)**

*-----------------------------------------------------------

Reviewer #3:

I have two broad concerns about the framing and conceptualization of the study.*

*First, the authors introduce and motivate the paper with a concern about the rising cost of insulin in the US. This is well-established and a genuine policy problem. But they seem to suggest—implicitly throughout and explicitly at times—that the rising cost of insulin is influenced by physician-industry relationships and the lack of transparency around these relationships. For instance, in the Discussion, they write that "Key factors related to the rise in insulin prices over the past two decades include…the lack of transparency in such financial relationships between physicians and industry for insulin." On the next page, they appear to fault the ADA Working Group for failing to mention "the industry marketing payments as factors in the escalating cost of insulin." 

However, the authors do not propose a mechanism by which industry payments to physicians, or the higher rates of prescribing that they might induce, would lead to higher drug prices. As such, the findings presented in the study, which show an association between industry payments and prescribing, do not appear to be plausibly linked to the policy problem of interest, namely, the rising cost of insulin. In this sense, the bookends of the paper seem to be dealing with a different problem (i.e., the cost of insulin) than the heart of the paper (i.e., determinants of insulin prescribing). The author should either reframe the Introduction and Discussion sections, or else specify the mechanism by which they believe industry payments to physicians affect insulin prices.*

**This is an excellent point. The goal of our paper was to investigate the association between industry payments to physicians and their claims and costs paid for claims of long-acting insulin, not the price of long-acting insulin. We think our findings of these associations generate a hypothesis that physician-industry financial relationships may increase overall costs of long-acting insulin for patients with diabetes, which works against the national effort to reduce patients’ financial burden of using insulin during this insulin affordability crisis. We apologize if this was not clear enough in our original submission. We have reframed the introduction and discussion sections of our revised manuscript to clarify this point.**

**“A study specific to insulin is important because Medicare Part D spending on insulin increased 840% from $1.4 billion in 2007 to $13.3 billion in 2017 [12,13] mainly owing to the rapidly increasing trajectory of insulin prices and the number of Medicare beneficiaries using insulin in the United States [13-16]. ……… Since physicians are the conduit between insulin suppliers and patients with diabetes, it is vital to also understand the dynamics of insulin prescribing within a national sample of physicians. Specifically, it is critically important to investigate whether the financial relationship between physicians and pharmaceutical companies is one of the upstream determinants of physicians’ prescription of long-acting insulin—the most commonly marketed, commonly used, and most costly type of insulin [9,19,20]” (Page 5, Paragraph 2)**

**“****The influence of the physician-industry financial relationship on clinical practice for diabetes care involving insulin has been of great concern under the regulating system for pharmaceutical manufactures setting insulin prices and direct competition in the insulin market [16,31].** **In this context, our findings focusing on long-acting insulin (which constitutes a large proportion of insulin prescriptions and expenditures in the US [20])** **generate a hypothesis that physician-industry financial relationship may increase overall costs of long-acting insulin for patients with diabetes, which works against the national effort to reduce the patients’ financial burden of using insulin.” (Page 15, Paragraph 1)** *Second, and somewhat relatedly, it isn't clear how we ought to interpret the implications of higher rates of long-acting insulin prescribing. Typically, in these sorts of studies, investigators are interested in the associations between industry payments and prescribing because of an underlying concern that payments might influence physicians to prescribe in ways that are clinically suboptimal or clinically equivalent but costlier. So, for instance, in DeJong et al. (JAMA IM 2016)—which this manuscript cites—the authors find that industry payments are associated with higher rates of prescribing brand name drugs when generics are available.

Do higher rates of long-acting insulin prescribing raise similar concerns? It isn't clear from the manuscript. One concern could be that physicians are prescribing an expensive drug, long-acting insulin, when a cheaper alternative would do. But in the Discussion, the authors say that "long-acting insulin is an essential drug and cannot be easily replaced for many patients" so that would seem not to be the case. Later, however, they say that detailing programs "focusing on a less expensive version of long-acting insulin" may be appropriate. If such alternatives exist, that should be stated earlier in the paper. More importantly, if there are both expensive and cheap versions of long-acting insulin, then this study's design would seem to be seriously flawed. In that case, the research question should be whether receipt of industry payments is associated with prescription of the more expensive type of long-acting insulin. But the question that this actually asked here is whether receipt of industry payments is associated with higher rates of prescribing any long-acting insulin.*

**This is also an important point. The current clinical guidelines indicate that long-acting insulin should be considered for patients with type 1 diabetes and patients with type 2 diabetes who do not achieve target glycemic control levels with oral agents and glucagon-like peptide 1 receptor agonists** (American Diabetes Association 2019)**. Given that long-acting insulin analogs are more expensive than oral agents and human insulin, which could be valid alternative options for some patients** (Cefalu et al. 2018; Lipska et al. 2017)**, and the pharmaceutical industry makes more profits through increased sales of long-acting insulins, there have been concerns about potential unnecessary prescriptions of long-acting insulins that are not supported by guidelines. Therefore, it is imperative to understand whether the financial physician-industry relationship is associated with increased claims and costs of long-acting insulin. We have clarified this point in the Introduction.**

**“Improving awareness about such associations for long-acting insulin analogs is needed because they are more expensive than other oral antihyperglycemic therapies and human insulin which could be alternative options for some patients with type 2 diabetes [9,10] and unnecessary prescriptions of long-acting insulins that are not supported by clinical guidelines [11] should be avoided to reduce the risk of hypoglycemia and save costs.” (Page 5, Paragraph 1)**

**Although a follow-on insulin glargine (Basaglar [Lilly]), a cheaper version of long-acting insulin, received FDA approval in December 2015, our data included only a limited number of physicians who prescribed this follow-on insulin. The use of such less expensive biosimilars is likely to increase in the near future, but pharmaceutical company marketing may work to maintain the market share of more expensive long-acting insulins. We argued the potential effectiveness of facilitating the use of such a newly less expensive version of long-acting insulin to reduce overall spending as a future strategy, but this argument was not directly supported by our data (without sufficient data on follow-up insulin or biosimilars). We have revised the manuscript to make a more balanced discussion based on our findings as follows:**

**“Although our data in 2017 included** **only a few physicians who prescribed Basaglar and was not sufficient to make an argument specific to this drug,** **future studies are needed to understand whether programs without conflicts of interest such as academic detailing [33] or other educational programs focusing on the follow-on insulin are effective to reduce the overall insulin costs by providing unbiased third-party information on long-acting insulins to physicians.” (Page 16, Paragraph 1)**

*I suppose another reason to be concerned about higher rates of long-acting insulin prescribing could be that physicians are overprescribing insulin. This is the underlying concern, for example, in recent studies of the association between industry payments and opioid prescribing. But that seems implausible in the case of insulin, and the authors provide no evidence to that effect.*

**We think it is possible that industry marketing to physicians introduces over-prescription of long-acting insulin; for example, instead of oral antihyperglycemic therapies for patients with type 2 diabetes. However, as with other studies on this topic (including opioid-related studies), we cannot differentiate necessary and unnecessary prescriptions using the Medicare Part D data. We have added this point in the revised manuscript.**

**“Although our data does not adjudicate the appropriateness of the prescription, it may also be possible that industry marketing payments introduce physicians’ preference of prescribing long-acting insulin rather than cheaper oral antihyperglycemic therapies” (Page 15, Paragraph 1)** *Perhaps some of these issues involving the availability of clinical alternatives and the implications of higher rates of long-acting insulin prescribing are obvious to specialists, but as this is a general medical journal, they would need to be more clearly spelled out.*

**Thank you for your suggestion. We have revised the paragraph clearly stating the clinical and policy implications of higher rates/costs of long-acting insulin prescriptions in the revised introduction and discussion section. (please see our response to your first comment).**

*Overall, these two issues—1) that the authors don't provide a plausible mechanism by which industry payments to physicians drive higher insulin prices, and 2) that the author don't otherwise clarify why higher rates of long-acting insulin are problematic—raise questions about the policy or clinical relevance of the study's findings.*

**We hope we have adequately addressed these two points (please see our response to your points above). Please let us know if anything remains unclear or needs to be improved.**

**References:**

American Diabetes Association. 2019. 9. Pharmacologic Approaches to Glycemic Treatment: Standards of Medical Care in Diabetes—2019. Diabetes Care 42:S90–S102; doi:10.2337/dc19-S009.

Anderson TS, Lee S, Jing B, Fung K, Ngo S, Silvestrini M, et al. 2020. Prevalence of Diabetes Medication Intensifications in Older Adults Discharged From US Veterans Health Administration Hospitals. JAMA Netw Open 3:e201511; doi:10.1001/jamanetworkopen.2020.1511.

Cefalu WT, Dawes DE, Gavlak G, Goldman D, Herman WH, Nuys KV, et al. 2018. Insulin Access and Affordability Working Group: Conclusions and Recommendations. Diabetes Care 41:1299–1311; doi:10.2337/dci18-0019.

Inoue K, Duru OK, Mangione CM, Tsugawa Y. 2020. Trends in Industry Payments to Physicians for Antihyperglycemic Therapies Between 2014 and 2018. J Gen Intern Med; doi:10.1007/s11606-020-06215-0.

Korenstein D, Keyhani S, Ross JS. 2010. Physician attitudes toward industry: a view across the specialties. Arch Surg 145:570–577; doi:10.1001/archsurg.2010.75.

Lipska KJ, Hirsch IB, Riddle MC. 2017. Human Insulin for Type 2 Diabetes: An Effective, Less-Expensive Option. JAMA 318:23–24; doi:10.1001/jama.2017.6939.

Sah S, Fugh-Berman A. 2013. Physicians under the influence: social psychology and industry marketing strategies. J Law Med Ethics 41:665–672; doi:10.1111/jlme.12076.

Sumarsono A, Everett BM, McGuire DK, Fonarow GC, Butler J, Pandey A, et al. 2020. Trends in Aggregate Use and Associated Expenditures of Antihyperglycemic Therapies Among US Medicare Beneficiaries Between 2012 and 2017. JAMA Intern Med 180:141–144; doi:10.1001/jamainternmed.2019.3884.

Tringale KR, Marshall D, Mackey TK, Connor M, Murphy JD, Hattangadi-Gluth JA. 2017. Types and Distribution of Payments From Industry to Physicians in 2015. JAMA 317:1774–1784; doi:10.1001/jama.2017.3091.

Williams R. 2012. Using the margins command to estimate and interpret adjusted predictions and marginal effects. Stata journal 45: 308–331.
